# Antibacterial Activity of Some Lactic Acid Bacteria Isolated from an Algerian Dairy Product

**DOI:** 10.1155/2009/678495

**Published:** 2009-08-12

**Authors:** Abdelkader Mezaini, Nour-Eddine Chihib, Abdelkader Dilmi Bouras, Naima Nedjar-Arroume, Jean Pierre Hornez

**Affiliations:** ^1^Laboratoire de Bio-Ressources Naturelles Locales, Université Hassiba Ben Bouali de Chlef, 02000 Chlef, Algeria; ^2^Laboratoire de Procédés Biologiques, Génie Enzymatique et Microbien (ProBioGEM) IUT A/Polytech'Lille, Université de Lille 1-Sciences et Technologies, Avenue Langevin, 59655 Villeneuve d'Ascq Cedex, France

## Abstract

In the present study, the antibacterial effect of 20 lactic acid bacteria isolates from a traditional cheese was investigated. 6 isolates showed antibacterial effect against Gram positive bacteria. *Streptococcus thermophilus* T2 strain showed the wide inhibitory spectrum against the Gram positive bacteria. Growth and bacteriocin production profiles showed that the maximal bacteriocin production, by *S. thermophilus* T2 cells, was measured by the end of the late-log phase (90 AU ml^−1^) with a bacteriocine production rate of 9.3 (AU ml^−1^) h^−1^. In addition, our findings showed that the bacteriocin, produced by *S. thermophilus* T2, was stable over a wide pH range (4–8); this indicates that such bacteriocin may be useful in acidic as well as nonacidic food. This preliminarily work shows the potential application of autochthonous lactic acid bacteria to improve safety of traditional fermented food.

## 1. Introduction

Lactic Acid Bacteria (LAB) isolated from dairy products have received increased attention as a potential food preservative due to their antagonistic activity against many food born pathogen such as *Listeria monocytogenes* [1]. LAB are widely distributed in the nature, they are typically involved in a large number of the spontaneous food fermentation, and they have been extensively studied [2]. Some members of LAB produce bacteriocins and bacteriocins-like substances which may inhibit growth of spoilage and pathogenic microorganisms [3]. Bacteriocins from LAB are bioactive peptides or proteins with antimicrobial activity toward Gram positive bacteria, including closely related strains and/or spoilage and pathogenic bacteria [4]. Bacteriocins are ribosomaly synthesized and extracellulary released bioactive peptides or peptide complexes which have bactericidal or bacteriostatic effect [5]. Use of either the bacteriocins or the bacteriocin-producing LAB like starter cultures for food preservation has received a special attention [6]. Moreover, bacteriocins are innocuous due to proteolytic degradation in the gastrointestinal tract [7, 8]. *S. thermophilus* is a lactic acid bacterium of major importance in food industry like the manufacture of yoghourt [9]. Some of *S. thermophilus* strains produce a bacteriocin named thermophilin which is active against several LAB and food spoilage bacteria such as *Clostridium sporogenes*. In view of its technological and biochemical properties the above bacteriocin can be considered as a potential bioprerservative [10]. Some of other LAB like *Enterococcus, Lactococcus,* and *Pediococcus* are also widely used as natural preservatives, due to the potential production of metabolites with antimicrobial activity such as organic acids, hydrogen peroxide, antimicrobial enzymes and bacteriocins [11].

The aim of the present study is to assess antimicrobial activity of some lactic acid bacteria strains isolated from traditional fermented dairy products prepared from raw milk, Raib, which is obtained after spontaneous curdling of raw milk within 24 to 36 hours at ambient temperature. In addition, preliminary investigations on a bacteriocin produced by *S. thermophilus* strain isolated in this work will be presented.

## 2. Materials and Methods

### 2.1. Isolation of Lactic Acid Bacteria

The bacterial strains used in this study were isolated from fermented traditional milk, Raib, manufactured without starter cultures. Samples were collected all over Chlef regions and obtained with collaboration of Bioressources research laboratory. LAB were isolated from Raib, by homogenizing 10 g samples of cheese in 90 mL saline solution and then plating suitable serial dilutions onto different media: BHI, MRS, and M-17 (Biokar Diagnostics, Beauvais, France). The plates were incubated aerobically at 30°C for 48 hours, and then several colonies were picked at random for identification. Cell morphology and Gram-staining reaction were examined by light microscopy, and the catalase activity was carried out. Phenotypic identification was based upon physiological and biochemical characteristics; sugar fermentation profile, in the API-20 Strep CH and API-50 CH fermentation, was carried out according to the manufacturer's instructions (bioMe’rieux, Marcy l’Etoile, France).

### 2.2. Detection of Antibacterial Activity

For detection of antagonistic activity, an agar spot test was used. The agar spot test was a modification of that described by Tomé et al. [12]. Overnight cultures, on MRS medium, of the strains to be tested for production of antimicrobial compound were centrifuged (10 minutes at 15000 g, 4°C). Cell-free supernatants were filtered across cellulose acetate filter (0.2 *μ*m) to remove residual cells.

An overnight culture (37°C) of the target strain was diluted in sterile Mueller-Hinton Medium, and 2 mL of *ca* · 10^6^ CFU mL^−1^ were spread on solid Mueller-Hinton medium. After 5 minutes of contact, the excess was removed and the Petri dishes were dried for 10 minutes. Samples (10 *μ*L) of filtered cell-free supernatants were spotted on the agar plate. The target strains used in this study are *Bacillus cereus* CIP 6624, *Bacillus subtilis* ATCC 6633, *Escherichia coli* CIP 35218, *Enterococcus faecalis* CIP 29212, *Listeria innocua* ATCC 51742, *Salmonella typhimurium* CIP 5858, *Staphylococcus aureus* CIP 29213, *and Staphylococcus epidermitidis* ATCC 14990.

### 2.3. Sensitivity of Bacteriocin to Enzymes, pH and Heat Treatment

The biochemical nature of the antibacterial agent was studied on both chloroform extract and cell-free supernatant; all the samples were incubated for 1 hour at 37°C before the antilisterial essay. The pH of cell-free supernatants was adjusted to 6.5 with NaOH (1 N) and then treated with catalase (Sigma; 500 IU mL^−1^). The cell-free supernatant was also submitted to heat treatment (60–95°C) and to several pH (4–8). The chloroform extract was treated with *α*-amylase (Sigma; 1 mg mL^−1^ 100 mM phosphate buffer, pH 6.9), *α*-chymotrypsin (Sigma, 1 mg mL^−1^, 0.05 M Tris–HCl buffer (pH 8.0)–0.01 M CaCl_2_), Pronase E (Sigma; 1 mg mL^−1^ in 100 mM Tris–HCl buffer, pH3), Proteinase K (Sigma; 1 mg mL^−1^ in 100 mM Tris–HCl buffer, pH 7.5), and Trypsin (Sigma, 1 mg mL^−1^ 50 mM Tris–HCl buffer pH 8.0). Prior to being assayed for bacteriocin activity, preparations containing pronase E were adjusted to pH 6.0. Neutralized cell-free supernatant neutralized cell-free supernatant treated with catalase, heat-treated supernatant, and chloroform extract were spotted against *L. innocua*. The enzymes were heat inactivated for 3 minutes at 100°C. For each test, untreated bacteriocin plus buffer, bacteriocin plus buffer treated 5 minutes at 100°C, buffer alone and enzymes solutions served as controls [13, 14].

### 2.4. Growth Kinetic and Bacteriocin Production

Growth experiments were performed in ErlenMeyer flask of 500 mL containing 250 mL of MRS broth (pH 6.5) at 37°C without shaking. An overnight pre-culture of *S. thermophilus* was used for the inoculation of the MRS broth at initial cell density of *ca* · 10^3^ CFU mL^−1^. At different time intervals, samples were removed from the culture and used for optical density measurement (660 nm), viable and cultivable count (CFU mL^−1^), extracellular pH measurements, and bacteriocin production. The antibacterial concentration of each sample was conducted with the critical method of dilutions [15]. The bacteriocin concentration Arbitrary Unit mL^−1^ (AU mL^−1^) was calculated as the inverse of the strongest dilution which induces the inhibition of *L. innocua*. All experiments were repeated at least three times. The experiments were repeated three times, and results are expressed as mean ± standard error to the mean.

### 2.5. Bacteriocin Extraction

The extraction was realized from cell-free culture supernatant of *S. thermophilus* obtained after centrifugation of overnight culture (20 minutes at 15000 g at 4°C). The extraction was performed according to Burianek and Yousef [16]. The culture supernatant (100 mL) was stirred vigorously for 20 minutes with chloroform (v/v) and transfer in separation funnel, the interface layer between the aqueous and organic phases, which contain bacteriocin, was harvested, and the residual chloroform was eliminated by speed vacuum (50 hours, Unique, Martinsried, Germany). Then bacteriocin activity was measured in the interface layer, aqueous and organic phases.

### 2.6. Plasmid Extraction

The plasmid extraction was performed from a cell pellet of an overnight culture of *S. thermophilus* (250 mL) using Miniprep Spin kit together with the corresponding buffers purchased from QIAGEN (Hilden, Germany). *S. thermophilus* plasmidic DNA analysis was performed by electrophoresis (1 hour, 100 V) using a 0.7% agarose gel dissolved in Tris 45 mM; Borate 45 mM; EDTA 1 mM; pH 8. The electrophoresis gels were analyzed under UV using Molecular Imager Gel Doc System (Bio-Rad, Hercules, USA).

### 2.7. HPLC Purification of Supernatant Chloroform Extract

The conditions for bacteriocin isolation were realized, through analytical RP-HPLC, on the chloroform extract. The liquid chromatographic system consisted of a Waters 600 E automated gradient controller pump module, a Waters Wisp 717 automatic sampling device, and a Waters 996 photodiode array detector. Spectral and chromatographic data were stored on a NEC image 466 computer. Millennium software was used to plot, acquire, and analyze chromatographic data.

All of the chromatographic processes were performed on an Uptisphere C_18_ column (150 mm × 4.6 mm, UP5ODB615QS, Interchim, Montluçon, France). The mobile phase was water/trifluoroacetic acid (1000 : 1, v/v) as eluent A and acetonitrile/trifluoroacetic acid (1000 : 1, v/v) as eluent B. The flow rate was 1 mL/min ^−1^. Samples were filtered through 0.22 *μ*m filters and then injected. The gradient applied was 0–50% (v/v) B over 100 minutes then 50%–100% (v/v) B over 5 minutes and 15 minutes at 100% (v/v) B. Online UV absorbance scans were performed between 200 and 300 nm at a rate of one spectrum per second with a resolution of 1.2 nm. Chromatographic analyses were completed with Millennium software [17].

## 3. Results

### 3.1. Antimicrobial Activity

Twenty LAB strains, isolated from Algerian dairy milk (Table 1), were screened for their antagonistic activity against *Listeria innocua, Enterococcus faecalis, Bacillus cereus, Bacillus subtilis, Staphylococcus aureus, Staphylococcus epidermitidis, Escherichia coli, and Salmonella typhimurium*. The results of Table 2 show that six isolates were active against one or more tested strains. However, *S. thermophilus* T2 strain showed a wide inhibitory spectrum against all the Gram positive target bacteria used in this study except against *Staphylococcus aureus* (Table 2). In addition, *S. thermophilus* T2 did not show any inhibitory activity against Gram negative bacteria used in this study: * Escherichia coli *and* Salmonella typhimurium.*

### 3.2. Nature of the Inhibitory Agent

Our results showed that the free-cell supernatant remained active, against sensitive target strains, even when the pH was adjusted to pH 7. However, when the cell-free supernatant and the chloroform extract were exposed to the proteolytic enzymes (Table 3) no inhibitory activity was observed against *Listeria innocua* by contrast to the control tests which showed an inhibitory activity against the target strain (Table 3). In addition, when the cell-free supernatant and the chloroform extract were exposed to the action of *α*-amylase and catalase similar inhibitory activity was measured when compared with the control test against *L. innocua*. These results suggest that the biochemical nature of the molecule produced by *S. thermophilus* is peptidic. Moreover, the antimicrobial activity appeared to be heat resistant. Thus, the inhibitory activity of the chloroform extract was still measured after a heat treatment of 30 minutes at 90°C. Our results showed also that in a range of pH 4–8 similar antibacterial activities of the chloroform extract were obtained against *L. innocua*.

### 3.3. Extraction of the Bacteriocin Produced by S. thermophilus

The extraction of the bacteriocin produced by *S. thermophilus* T2 strain from culture supernatant was realized with chloroform, a water-immiscible solvent. The method used concentrates the bacteriocin at the interface between chloroform and the aqueous culture of the producing bacterium. We demonstrated that no bacteriocin activity was detected in the solvent phase (data not shown). In addition, the precipitate at the interface between the chloroform and culture supernatant fluid contained most of the bacteriocin activity in the mixture. The precipitate at the interface was harvested, and the residual chloroform was eliminated by speed vacuum. After HPLC reversed-phase chromatography, bacteriocin activity was associated with two peaks eluting at 17 minutes and 110 minutes (Figure 3). These results showed that the antibacterial activity of *S. thermophilus* T2 could be associated with two molecules which present different hydrophobicity.

### 3.4. Growth Kinetics and Bacteriocin Biosynthesis

**Figure 1:**
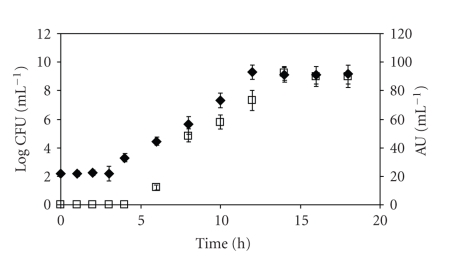
Growth kinetic and bacteriocin production by *S. thermophilus*. The growth was performed at an initial pH of 6.5, at 37°C without shaking. (◆) growth kinetic. (□) bacteriocin production. The experiments were repeated three times and results represent the mean ± standard error to the mean.

Growth and bacteriocin production of *S. thermophilus* was studied in MRS broth at 37°C at pH 6.5. Under these conditions bacteriocin activity was detected at 4 hours of incubation at the beginning of the exponential phase, at a cell concentration of *ca* · 10^4^ CFU mL^−1^ (12 AU mL^−1^). The results of Figure 1 showed that bacteriocin production increases with the increase of cell concentration to reach a maximum of 90 AU mL^−1^ with a bacteriocin production rate of 9.3 (AU mL^−1^) h^−1^. This concentration was reached between 12 and 14 hours of incubation at 37°C. During the stationary phase both bacteriocin concentration and the cell concentration remained at a steady state (Figure 1). Antibacterial activity decreased after 24 hours of incubation after having reached maximum levels after 14 hours of incubation (data not shown).

### 3.5. Plasmid Content

**Figure 2:**
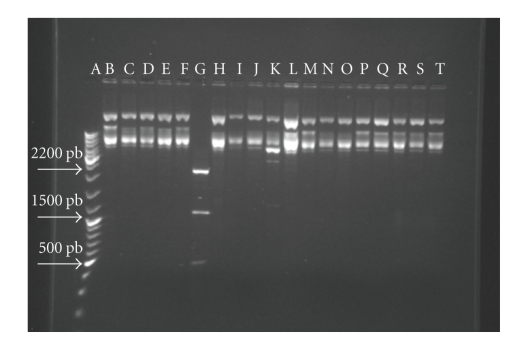
Agarose gel electrophoresis of plasmid from *S. thermophilus* digested by various restriction endonucleases: (A) O’Gene Ruler control (B); *Ava*III (C); *Bam*HI; (D) *Bgl*II; (E) *Bst*EII; (F) *Eco*RI; (G) *Dra*I; (H) *Eco*RV; (I) *Hin*dIII; (J) *Nde*I; (K) *Mfe*I; (L) *Pst*I; (M) *Pvu*II; (N) *Sac*I; (O) *Sca*I; (P) *Sph*I; (Q) *Xho*I; (R) *Aat*II; (S) *Aha*III; (T) *Nco*I.

**Figure 3:**
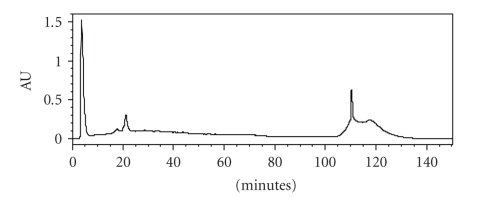
Elution pattern of chloroform extract from *S. thermophilus* T2 strain by reversed-phase high-performance liquid chromatography.

The genes encoding for bacteriocin are either chromosomic or plasmidic [18, 19]. The aim for this preliminary investigation is to assess the presence of plasmid in *S. thermophilus* cells. The analysis of the plasmidic DNA extraction showed that *S. thermophilus* seems to have a single plasmid as shown in Figure 2. In addition, *Dra*I fragmentation pattern of the *plasmid* resulted in three *restriction fragments* with approximately 2.2 kb, 1.5 kb, and 0.5 kb. Thus the size of the plasmid could be of at least 4.2 kb. This preliminary result is of importance since in many lactic acid bacteria bacteriocins and carbohydrate fermentation exopolysaccharide production and antiphage mechanisms are carried by the same plasmid as reported previously by Martinez-Bueno et al. [20] and Turgeon and Moineau [21].

## 4. Discussion

Bacteriocins from lactic acid bacteria are of importance in bioconservation of various foods. Moreover, the use of more than one LAB bacteriocin as a combination of biopreservative may have major applications in improving food safety [1]. In the present study, the inhibitory effect of the cell-free filtrates of each of the 20 isolates was evaluated. Antimicrobial activity was observed for 6 isolates, and only against Gram positive bacteria. The biochemical nature of the antibacterial molecule produced by *S. thermophilus* T2 was studied in both the cell-free supernatant and the chloroform extract. Our results showed that the molecule, produced by *S. thermophilus*, is peptidic since the antibacterial activity of the molecule was lost after digestion with proteolytic enzymes. However, the neutralization (pH 7) and addition of catalase or *α*-amylase to the cell-free supernatant did not result in the loss of the antilisterial activity. Our results showed also that the bacteriocin produced by *S. thermophilus* is heat stable (up to 30 minutes at 95°C); these results are similar to what has been reported for thoenicin [22]. In addition, the bacteriocin was stable over a wide pH range, this indicates that such bacteriocin may be useful in acidic as well as nonacidic food; similar pH stability results have been reported for propionicin PLG1 [14]. Growth and bacteriocin production profiles showed that the maximal bacteriocin production was measured by the end of the late-log phase. The level of production remained at a steady state during the stationary phase; similar results were obtained by Ivanova et al. [23]. However, bacteriocin production decreases after 24 hours of incubation after having reached maximum levels after 14 hours. This reduction could be a result of the inactivation of bacteriocin by extracellular proteases.

Preliminary characterization of the bacteriocin produced by *S. thermophilus* T2 was realized in the present study. It was found that the bacteriocin inhibits closely related Gram positive strains like *Listeria innocua* and *Enterococcus faecalis*. Activity against Gram negative was rarely reported for bacteriocin [24, 25]. Active substance from culture supernatant of *S. thermophilus* T2 was obtained according to the procedure described by Burianek and Yousef [16]. Chloroform was added to the cell-free supernatant in a separator funnel, the bacteriocin was concentrated at the interface between chloroform and the aqueous phase. This method effectively recovers higher bacteriocin yield and results in relatively clean preparations. Recovery of bacteriocin by the chloroform extraction was 10-fold higher when compared with ammonium sulphate precipitation (data not shown). The chloroform extraction procedure saves time, and it is easy to perform. This study allowed to underline the presence of at least one plasmid, of 4.2 kb as reported for many strains of *S. thermophilus* [21].

In conclusion, the study of autochthonous LAB will help to select the best candidates for improving the microbiological safety of traditional food products such as Raib and may increase their shelf life. Such a collection could be used for construction of specific starter cultures for fermented food products.

## Figures and Tables

**Table 1:** Lactic acid bacteria isolated from traditional dairy product (Raib).

Strain	Source	Growth medium
*Lactococcus lactis* S1	Raib	MRS
*Lactococcus lactis* S2	Raib	MRS
*Lactococcus lactis* S3	Raib	MRS
*Lactococcus lactis* S4	Raib	MRS
*Lactococcus lactis* S5	Raib	MRS
*Lactococcus lactis* S6	Raib	MRS
*Lactococcus lactis* S7	Raib	MRS
*Lactococcus lactis* S8	Raib	MRS
*Lactococcus lactis* S9	Raib	MRS
*Lactococcus lactis* S10	Raib	MRS
*Lactococcus lactis* S11	Raib	MRS
*Lactococcus lactis* S12	Raib	MRS
*Lactococcus lactis* S13	Raib	MRS
*S. thermophilus* T1	Raib	MRS
*S. thermophilus* T2	Raib	MRS
*S. cremoris* R1	Raib	MRS
*S. cremoris* R2	Raib	MRS
*S. cremoris* R3	Raib	MRS
*Lactococcus diacetylactis* V1	Raib	MRS
*Lactococcus diacetylactis* V2	Raib	MRS

**Table 2:** Antibacterial spectrum of the cell-free supernatant of the six lactic acid bacteria isolated from the traditional dairy product (Raib).

Strain	Strains inhibited
*Lactococcus lactis* S1	*Listeria innocua*, *Enterococcus faecalis *
*Lactococcus lactis* S2	*Listeria innocua *
*Lactococcus lactis* S7	*Listeria innocua*, *Enterococcus faecalis Bacillus cereus *
*Lactococcus lactis* S9	*Listeria innocua*, *Bacillus cereus *
*S. thermophilus* T2	*Bacillus cereus, Bacillus subtilis, Listeria innocua, Enterococcus faecalis, and Staphylococcus epidermitidis*
*S. cremoris* R3	*Enterococcus faecalis*, *Bacillus cereus *
*Lactococcus diacetylactis* V1	*Enterococcus faecalis *

**Table 3:** Effect of different treatments on cell-free supernatant and chloroform extract of *S. thermophilus* T2. Relative activity was measured by an agar diffusion test against *Listeria innocua*. (−): no inhibition; (+): slight inhibition; (++): moderate inhibition; (+++): strong inhibition.

Treatments	Relative activity
Enzymatic treatments	

Proteinase K	−
Pronase E	−
*α*-chymotrypsin	−
Trypsin	−
*α*-amylase	++
Catalase	++
Control	+++

pH treatments	

4	+++
5	+++
6	+++
7	+++
8	+++
Control	+++

Heat treatments	

60°C	+++
70°C	+++
80°C	++
90°C	++
95°C	+
Control	+++
